# Supplementary material for: Functional Analysis of Membrane-Associated Scaffolding Tight Junction (TJ) Proteins in Tumorigenic Characteristics of B16-F10 Mouse Melanoma Cells
Source: Int J Mol Sci. 2024 Jan 9;25(2):833. doi: 10.3390/ijms25020833 (PMC10815660; doi:10.3390/ijms25020833)
Supplement: Supplementary file 1 [file ijms-25-00833-s001.zip › ijms-2755227-supplementary.pdf]

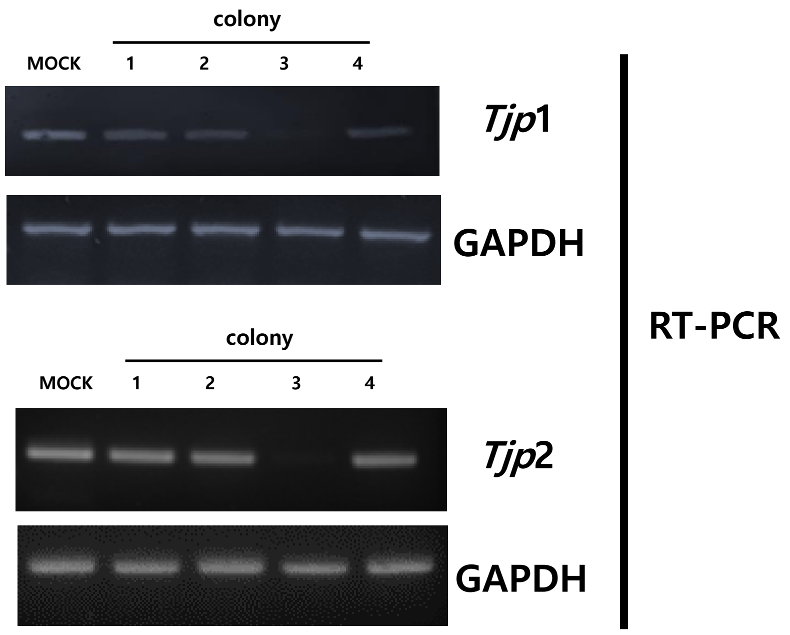

Supplemental Figure S1. After selecting the CRISPR/Cas9 system-transfected cells, we confirmed the Tjp1 and Tjp2 knockout clones using RT-PCR.

TGTGTTGGGGAAATGTGCTGAGCTCATGAGC
TCCTACCAAGTAAGTTGTGCATTAGTTTGTGAT
AAATAAATGTATCACTTAGTATGTTGGGACAAT
TACACATATTGATAATGATGCACTGTCAAATTT
TGATTAAGGAATGAGGTCAGGCTTGTGAGTTT
GTTGTGGTTTTGGTTACAAAGTGTTCATAAGAT
ATGTGGTATCTTTAGAGTATAGTTCTAATCCTT
CATTATTTTACACAAGTATTAATTATCACCTGG
AAAATTGTTATAAATTCTGGAAGGATTTAGTAA
ATTTACTGTTTAGCCAGATCTGTTTTATTAAGA
GAGTGGAACACAGCCATTTTGGAACTGTATTG
GTGTGATTACTCTATTATCTCCTTTGTATTTACA
TAAGTCAAGGGAAATGTATGTTTCTCCTATGA
GTGGAGAGTATATGACTATCGTTCAGTTTATTA
TTTCTTTTTGTTTGTAG

Intron

ACTATCCGAAGGAAGA
AGAAAGTTCAGATCCCTGTAAGTCACCCAGAT
CCTGAGCCGGTGTCTGATAATGAAGACGATAG
TTATGACGAAGAAGTGCATGACCCAAGAGCTG
GCCGCGGTGCTTTAGCGAACAGAAGGAGCGA
GAAGAGCTGGGCAAGGGATAGGAGTGCAAGC
AGGGAGAGGAGCCTGTCCCCTCGCTCGGACA
GGCGGTCCGTGGCCTCCAGTCAGCCCGCAAA
GCCCACCAAGGTCACACTGGTGAAGTCTCGG
AAAAATGAAG

Exon5

GTATCACTGACTCTCACTGTTCT
CACGTGAGACTTTTAAAGCTAGTTTAATAAGA
AATACAATGACCATCTTATCTGGTTTTTTTCCAT
TTCTCTTTCTAGTCATGTTATCTTGCTAAGAGTC
AAATCCTGGTAAGGTAGACATTGGGATATTTT
GGAACCTTTTCTCAAATCATTTTGTTGGTGCAA
ACTTGTAGTTGAATTTTGGTTTTATTGGAACAG
GAATAGCCAAATTCAAATTGTGCT**AGCAAGA
AATGTTGGGCCAG**

Intron

GTTGTCAGGGATACAGATGTTTAAGGTTTGTTGT
AGGTTTGTTTTGGTTTGTTTTTGTATGACCCTCCC
ACCCTCCTCACACACACTGAGACTAGAGACTGA
CAGTTTTAATTAAAACTGGATCCAGGCCCACTG
CCCTCCCAGGTGTGTCTCTGCCGCCCTGCATCAT
CTACTCTATTTGTCTGAATAG

Intron

GTGGTCAAGAGAC
CCCGGAAGGTTCAGGTGGCCCCACTGCAAGGC
AGCCCCCCTCTCAGTCATG**ACGACCGAGGTTT
TGAAGTG**ATTGAAGAATTTGATGGCAGAAGTTT
CCGCAGCGGCTACAGTGAGAGGAGTCGGCACA
GCAGCCACGACATGCTCAGCCACAGCTGGGAG
GGCAATAGGGAGAGGGGGCGACCCCATCAGC
GCACACAGAGCCGGGAGCGGGAGCGCAGCCG
TGGCAGGAGCCTGGAACGGGGCCTGGACCAG
GAAGACTATGGGCGCAGCCGTGAGCGCAGCCG
TGGCCGGAGCCTGGAGCGCGGGCTGGACCGC
GACTTTGTGTCCAGGGACCACAGCCGTGGCCG
TAGCATCGACAGGGACTACGACCGAGACTATGA
GCGCTCCTATCACGAAGCTTATGAACCCGACT
ACGGAGGCGGCTACAGCCCATCATATGACCGTA
GAGCCCATCCAGAGACCCGCTATGAACGGAGC
CGCAGCCGAGAACACCTACGTTCCCGAAGCCC
CAGCCCCGAGTCTAGGTCACGG**CATGAACACA
AGGGCCAACA**TGATCCCGACAGGCCCATCGG
GGTCCTTCTAACCAAAAGCAAAGCAAATGAAG

Exon5

GTAGGCACACATGGTGTGGGGATACAAGCTGTA
CCTGCAGCAGCATCACCTGGTGCTCGCCTGGCA
CCTGCCAGTGTTCAGCGTCAACCCGAACTGGCC
AGCACCTATGGACTCATTTAGTTATCACTGCCAT
AAGAGAGTCAGAGATGCAGAAGGGTCCATTGA
TCTGGACGTAGTTGTCCCAGTGGCAAAGCCAGG
TCTTAAGAGATGTTCTTAACTT

Intron

Sequence : Primer
Red : gRNA

Supplemental Figure S2. Amplifying the gRNA-selected region using specific primers.
